# Annual epidemiological and health insurance disease burden of hip osteoarthritis in Hungary based on nationwide data

**DOI:** 10.1186/s12891-024-07513-y

**Published:** 2024-05-23

**Authors:** Luca Fanni Kajos, Bálint Molics, Diána Elmer, Dalma Pónusz-Kovács, Bettina Kovács, Lilla Horváth, Tímea Csákvári, József Bódis, Imre Boncz

**Affiliations:** 1 Institute for Health Insurance, Faculty of Health Sciences, University of Pécs, Pécs, Hungary; 2 Doctoral School of Health Sciences, Faculty of Health Sciences, University of Pécs, Pécs, Hungary; 3 National Laboratory On Human Reproduction, University of Pécs, Pécs, Hungary; 4 Institute of Physiotherapy and Sport Science, Faculty of Health Sciences, University of Pécs, Pécs, Hungary; 5 Department of Obstetrics and Gynaecology, Clinical Centre, Medical School, University of Pécs, Pécs, Hungary

**Keywords:** Hip osteoarthritis, Disease burden, Health insurance, Financing, Acute inpatient care, Outpatient care

## Abstract

**Background:**

Health services utilization related to hip osteoarthritis imposes a significant burden on society and health care systems. Our aim was to analyse the epidemiological and health insurance disease burden of hip osteoarthritis in Hungary based on nationwide data.

**Methods:**

Data were extracted from the nationwide financial database of the National Health Insurance Fund Administration (NHIFA) of Hungary for the year 2018. The analysed data included annual patient numbers, prevalence, and age-standardized prevalence per 100,000 population in outpatient care, health insurance costs calculated for age groups and sexes for all types of care. Patients with hip osteoarthritis were identified using code M16 of the International Classification of Diseases (ICD), 10th revision. Age-standardised prevalence rates were calculated using the European Standard Population 2013 (ESP2013).

**Results:**

Based on patient numbers of outpatient care, the prevalence per 100,000 among males was 1,483.7 patients (1.5%), among females 2,905.5 (2.9%), in total 2,226.2 patients (2.2%). The age-standardised prevalence was 1,734.8 (1.7%) for males and 2,594.8 (2.6%) for females per 100,000 population, for a total of 2,237.6 (2.2%). The prevalence per 100,000 population was higher for women in all age groups. In age group 30–39, 40–49, 50–59, 60–69 and 70 + the overall prevalence was 0.2%, 0.8%, 2.7%, 5.0% and 7.7%, respectively, describing a continuously increasing trend. In 2018, the NHIFA spent 42.31 million EUR on the treatment of hip osteoarthritis. Hip osteoarthritis accounts for 1% of total nationwide health insurance expenditures. 36.8% of costs were attributed to the treatment of male patients, and 63.2% to female patients. Acute inpatient care, outpatient care and chronic and rehabilitation inpatient care were the main cost drivers, accounting for 62.7%, 14.6% and 8.2% of the total health care expenditure for men, and 51.0%, 20.0% and 11.2% for women, respectively. The average annual treatment cost per patient was 3,627 EUR for men and 4,194 EUR for women.

**Conclusions:**

The prevalence of hip osteoarthritis was 1.96 times higher (the age-standardised prevalence was 1.5 times higher) in women compared to men. Acute inpatient care was the major cost driver in the treatment of hip osteoarthritis. The average annual treatment cost per patient was 15.6% higher for women compared to men.

## Background

Osteoarthritis (OA) is one of the most common joint disorders worldwide with a substantial and increasing social and economic burden for both the patient and the healthcare system [1–4]. It is a major cause of musculoskeletal disability and one of the most frequent causes of disability leading to limitation of daily living activities in the adult population [5–7]. In terms of global disease burden, osteoarthritis ranks 11th out of 291 diseases. However, its ranking varies by region, being the 6th in East Asia and high-income East Pacific countries, 10th in North America, 7th in Eastern Europe, and 13th in Western Europe [5, 8–10].

Osteoarthritis can occur in any joint in the body, but it typically affects the knees, hips, hands, spine, facet joints and feet [1, 11]. Osteoarthritis of the hip is less common than osteoarthritis of the hand or knee [1, 8, 12]. According *Oliveria *et al*. w*hile the incidence of knee osteoarthritis was the highest (240/100,000 person-years), hip osteoarthritis had the lowest incidence at 88/100,000 person-years [13]. A disease burden study in 2016 reported an incidence rate for hip osteoarthritis of 1.1/1,000 patients per year for Peruvian patients [14]. Compared to previous studies, a Spanish study reported higher incidence rates for hip OA in more than 3 million subjects (2.1/1,000 person-years) [15]. According to a recent global burden of disease study, between 1990 and 2019, the age-standardised incidence rate of hip osteoarthritis rose from 17.02 to 18.70 per 100,000 persons The prevalence outcomes for hip osteoarthritis differ significantly in the studies but based on a recent Global Burden of Disease Study (2022), the estimated prevalence was 0.4% in 2019 [16].

Osteoarthritis is not only a leading cause of disability among older adults, but also imposes a significant societal cost. Previous estimates of the economic burden of musculoskeletal disorders suggest that the economic impact of osteoarthritis is equivalent to two percent of GDP in industrialized countries, of which the largest direct costs are medication and surgery [17, 18]. According to a disease burden study for 2013, total annual costs per patient with knee and hip OA were ranged from 0.7 to 12 k€, direct costs from 0.5 to 10.9 k€ and indirect costs from 0.2 to 12.3 k€. The weighted average annual costs per patient were 11.1 for total, 9.5 for direct and 4.4 k€ for indirect costs [19]. According to another study, the cost of hip osteoarthritis-related care in the year prior to hip replacement surgery was 500–800$ per patient [20]. The most expensive treatment for OA is joint replacement surgery [5, 21]. Between 2003 and 2013, the rate of hip replacements in Australia increased from 88 to 129 per 100,000 population, at an estimated total expenditure of 364 million $AUD to 625 million $AUD per year [3].

Disease burden analyses have become widespread in academic research and policy-making over the last two decades. Disease burden analyses provide a quantitative overview of the health, social and economic implications associated with a particular disease or health condition [22, 23]. Understanding the relative costs and effectiveness of available treatments and preventive measures is of major importance. To enable stakeholders to plan treatments and surgical procedures for hip osteoarthritis, it is first important to understand its prevalence and costs [17].

As the publications show, hip osteoarthritis is a highly prevalent chronic joint disorder that constitutes a significant and growing burden on patients, health care systems and the broader society. Prevention, effective and cost-efficient care are of paramount importance in addressing the challenges of hip osteoarthritis. The aim of the study was to determine the annual health insurance treatment cost and the epidemiological disease burden of hip osteoarthritis in Hungary on nationwide data for the year 2018.

## Methods

Data were derived from the nationwide financial database of the National Health Insurance Fund Administration (NHIFA) of Hungary for the year 2018. Patients with hip osteoarthritis were identified with the code M16 of the International Classification of Diseases (ICD), 10th revision [24].

The data from the NHIFA database covers all the publicly financed health care providers. The main variables differ by sub-budget or type of care. The core variables are sex, age, place of residence, diagnosis (according to ICD 10th revision), medical procedures, number of patients, number of cases, and costing units (outpatient care, residential care, CT, MRI: fee-for-service based on ICPM (International Classification of Procedure in Medicine) codes; acute inpatient care: DRG (diagnosis-related group) cost weight; chronic and rehabilitation inpatient care: nursing days; home care: visits). The data was aggregated and provided in aggregated form by NHIFA according to the data request algorithm. No individual patient level data was requested and received during the data request, therefore anonymisation was not required.

To investigate the epidemiological and health insurance disease burden, all health insurance treatment categories were analysed, including general practitioner (GP) care, home care, outpatient care, acute and chronic and rehabilitation inpatient care, patient transportation, medical imaging (computed tomography [CT], magnetic resonance imaging [MRI], positron emission tomography [PET]), laboratory diagnostics, pharmaceuticals, and medical aids. Only the records for the item “Main diagnosis justifying care 3” were analysed for the data reported within the acute, and chronic and rehabilitation inpatient care.

Health insurance costs were calculated differently depending on the type of care [25]. GP care financing has several components, including a capitation fee, a fixed fee and quality indicators. For these calculations, we divided the total annual GP expenditure by the number of cases. In the case of outpatient care, the International Classification of Procedure in Medicine (ICPM) by World Health Organization (WHO) coding system was used and the fee-for-service method was applied. The same methodology was used for residential care, laboratory diagnostics and CT. The funding of acute inpatient care was calculated according to the cost-weights based on DRG (diagnosis-related group) (weight*198,000 HUF). A daily fee was applied for chronic and rehabilitation inpatient care. When financing home care, the fee was determined by type of visit. To define the patient transportation fee, we divided the total NHIFA transportation expenditure by the number of cases. For pharmaceuticals and medical aids, we only examined prescription drugs and medical aids covered by NHIFA coded M16 ICD (we did not include co-payment, costs paid by the patient).

In order to report the transferability of costs we used the following methods. The cost related to hip osteoarthritis (M16 ICD code) for all types of health care provisions was compared with the total nationwide health insurance cost (NHIFA) for all diseases: health insurance cost of osteoarthritis was divided by the total nationwide health insurance cost for all diseases. Therefore, we obtained the annual market share of hip osteoarthritis as a proportion of total nationwide NHIFA expenditure for each type of health care provisions.

### Statistical analysis

According to epidemiological aspects, the analysed data included annual patient numbers, prevalence, and age-standardized prevalence per 100,000 population in outpatient care, health insurance costs calculated for age groups and sex across all types of care. To calculate the prevalence and to compare it according to gender and age groups, we used the Hungarian population database of the Hungarian Central Statistical Office (HCSO) for the year 2018. The number of patients in outpatient care was divided by the population number and then multiplied by 100,000 to obtain the prevalence per 100,000 persons, also broken down by age group and sex. Age-standardised prevalence rates were calculated using the European Standard Population 2013 (ESP2013) [26]. The health insurance disease burden was examined included annual health insurance costs, patient numbers and cost distribution calculated for age groups (30–39; 40–49; 50–59; 60–69; 70 + years) and sex. To eliminate possible duplications between the different sub-budgets of the health insurance fund the most costly acute inpatient fund was used to determine the annual health insurance expenditure and per capita expenditure. Health insurance expenditure was expressed at the 2018 annual average exchange rate of the National Bank of Hungary: 1 Euro (EUR) equals to 318.87 Hungarian Forint (EUR), in order to be also comparable internationally. No costs were available for the ambulance service and no patient data were reported for PET. Ethical approval was not required for this database analysis.

## Results

Table 1 shows the number of patients diagnosed with hip osteoarthritis and the corresponding annual health insurance treatment costs reported in 2018, according to the type of care provided. The highest national patient numbers were found in outpatient care (69,311 men, 148,375 women, in total 217,686 patients), followed by general practice care (63,551 men, 138,030 women, in total 201,581 patients), and pharmaceuticals (29,071 men, 68,618 women, in total 97,689 patients), respectively.

**Table 1:** Number of patients, number of cases, natural units and health insurance expenditure for hip OA by type of care (NHIFA, 2018)

Type of care	Number of patients			Number of cases			Natural units				NHIFA expenditure (EUR)		
	**Male**	**Female**	**Total**	**Male**	**Female**	**Total**	**Natural unit**	**Male**	**Female**	**Total**	**Male**	**Female**	**Total**
General practitioner care	63 551	138 030	201 581	239 265	527 214	766 479	*--*	*--*	*--*	*--*	528 699 €	1 164 973 €	1 693 672 €
Home care	2 071	3 644	5 715	4 276	7 862	12 138	Number of visits	11 242	21 138	32 380	352 258 €	654 760 €	1 007 018 €
Patient transportation	2 079	4 952	7 031	8 850	21 152	30 002	Kilometres	210 107	474 489	684 596	166 047 €	362 768 €	528 815 €
Ambulance service	50	115	165	52	127	179	Kilometres	2 108	3 946	6 054	*--*	*--*	*--*
Outpatient care	69 311	148 375	217 686	313 309	707 370	1 020 679	Point	402 177 194	938 092 890	1 340 270 084	2 278 940 €	5 341 397 €	7 620 338 €
Residential care	94	269	363	291	846	1 137	Point	595 012	1 715 772	2 310 784	3 443 €	9 997 €	13 440 €
Laboratory diagnostics	4 999	7 952	12 951	7 973	11 976	19 949	Point / Tests	19 468 949/77 092	30 489 843/121 286	49 958 792/198 378	38 138 €	60 071 €	98 209 €
CT, MRI	1 219	1 845	3 064	1 929	2 897	4 826	Point	17 635 117	26 167 982	43 803 099	100 545 €	149 189 €	249 734 €
PET	0	0	0	0	0	0		-	-	-	0 €	0 €	0 €
Acute inpatient care	4 291	6 378	10 669	4 677	6 952	11 629	DRG-weight	19 444	29 723	49 167	9 758 396 €	13 652 299 €	23 410 695 €
Chronic and rehabilitation inpatient care	2 356	5 475	7 831	2 517	5 890	8 407	Nursing day	45 363	105 980	151 343	1 275 394 €	2 998 118 €	4 273 512 €
Pharmaceuticals	29 071	68 618	97 689	148 764	378 188	526 952	*--*	*--*	*--*	*--*	295 076 €	802 636 €	1 097 712 €
Medical aids	6 924	15 597	22 521	10 850	22 691	33 541	*--*	*--*	*--*	*--*	767 380 €	1 553 180 €	2 320 559 €
*Total*	***--***	***--***	***--***	***--***	***--***	***--***	***--***	***--***	***--***	***--***	***15 564 315 €***	***26 749 388 €***	***42 313 704 €***

Regarding the sex distribution of patients in the different NHIFA funds, we found that the proportion of female patients was higher in each category: in outpatient care 31.8% male and 68.2% female, in general practice care 31.5% male and 68.5% female, and in pharmaceuticals 29.8% male and 70.2% female, respectively. This difference was most balanced for laboratory diagnostics, CT-MRI funds, and acute inpatient care.

**Fig. 1:**
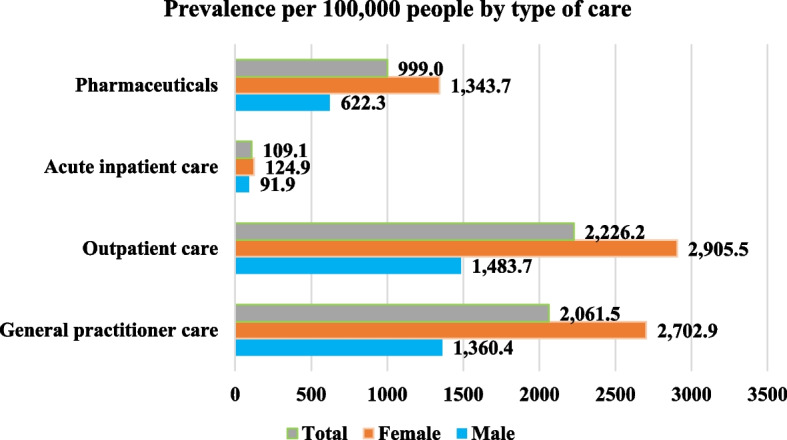
Prevalence per 100,000 people according to type of care in Hungary, 2018

Figure 1 shows the prevalence of the most frequent types of care for females and males. As we can see, the prevalence rates for each type of care differed considerably. While acute inpatient care had the lowest prevalence (due to lower patient numbers), outpatient care showed the highest prevalence. In all cases, females had a higher prevalence than males.

**Fig. 2:**
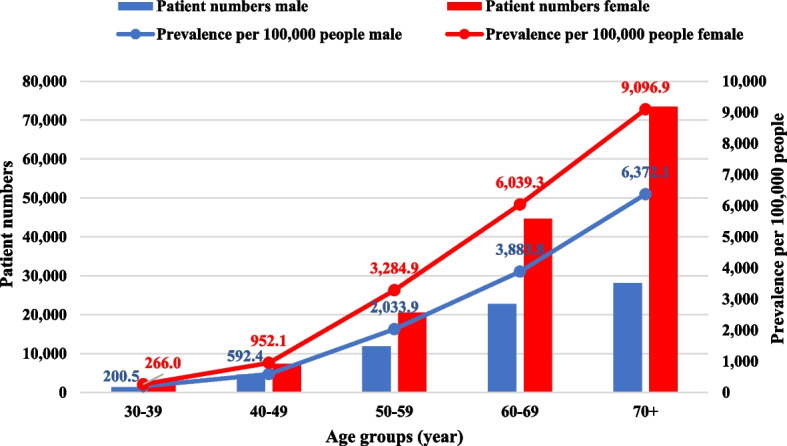
Total patient number and prevalence per 100,000 people according to age groups and sex based on outpatient care in Hungary, 2018

Figure 2 illustrates the number of patients with hip osteoarthritis by age and sex and the prevalence per 100,000 persons according to data from outpatient care, which had the highest number of patients. Examining the utilisation of care by age and sex, we found that the number of patients was higher for both sexes with increasing age, with considerably higher rates for females. In terms of outpatient care, in age groups 30–39, 40–49, 50–59, 60–69 and 70 + , the number of female patients were 1,730, 7,351, 20,506, 44,646, and 73,479, respectively, while these numbers among males were 1,350, 4,657, 11,858, 22,729, and 28,142 persons. Regarding the total number of patients, both male and female patients over 70 years had the highest patient numbers, however, the number of female patients was 2.5 times higher than that of male patients. At this age, the number of male patients was considerably lower compared to females, but the prevalence rate remained high. This ratio can be partly explained by the lower average life expectancy of males. According to the OECD (Organisation for Economic Co-operation and Development) database, the average life expectancy at birth in Hungary in 2018 was 72.7 years for males and 79.6 years for females (a difference of 6.9 years). Life expectancy over the age of 65 was 14.6 years for males and 18.5 years for females in 2018 (a difference of 3.9 years) [27]. The extremely high number of female patients over 70 accounted for 49.5% of all female patients and 33.75% of all patients of both sexes.

When we compared the acute inpatient patient numbers with the chronic and rehabilitation inpatient patient numbers, we observed that females over the age of 30 had a higher rate of rehabilitation utilisation than males. In age group 30–39, 40–49, 50–59, 60–69 and 70 + , the rehabilitation utilisation rate of female patients was 56.10%, 45.93%, 68.10%, 78.69%, and 98.20%, respectively, while among males, these rates were 34.04%, 33.62%, 42.44%, 53.17%, and 66.22%.

Based on patient numbers related to outpatient care, the prevalence per 100,000 was 1,483.7 patients (1.5%) among males, 2,905.5 patients (2.9%) among females, in total 2,226.2 patients (2.2%). Prevalence per 100,000 population was higher for women across all age groups (Fig. 2). In the age groups 30–39, 40–49, 50–59, 60–69, and 70 + , the prevalence of female patients was 0.3%, 1.0%, 3.3%, 6.0%, and 9.1%, respectively, while among males, these percentages were 0.2%, 0.6%, 2.0%, 3.9%, and 6.4%. The overall prevalence by age group was 0.2%, 0.8%, 2.7%, 5.0%, and 7.7%, respectively, indicating a continuously increasing trend.

**Fig. 3:**
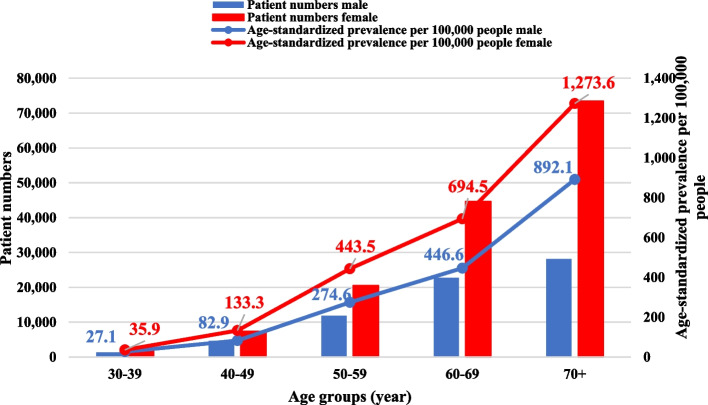
Total patient number and age-standardised prevalence per 100,000 people according to age groups and sex based on outpatient care in Hungary, 2018

Age-standardisation of the prevalence data was conducted using the European Standard Population 2013 (ESP2013) [26] (Fig. 3). The age-standardised prevalence was 1,734.8 (1.7%) for males and 2,594.8 (2.6%) for females per 100,000 population, for a total of 2,237.6 (2.2%). The age-standardised prevalence showed a similar pattern to the non-standardised calculation between men and women, with higher prevalence rates observed among women. Age-standardised prevalence for women in the age groups 30–39, 40–49, 50–59, 60–69, 70 + were 35.9, 133.3, 443.5, 694.5, 1,273.6, respectively, while for men, these rates were lower, at 27.1, 82.9, 274.6, 446.6, and 892.1, respectively.

The annual health insurance costs associated with hip osteoarthritis are summarised in Table 1, categorised by health insurance fund. In 2018, the NHIFA spent 42.31 million Euros (EUR) toward the treatment of patients with hip osteoarthritis. The various NHIFA expenditure categories showed considerable discrepancies among different types of care. Acute inpatient care (23.41 million EUR), outpatient care (7.62 million EUR), and chronic and rehabilitation inpatient care (4.27 million EUR) were the main cost drivers. The highest expenditure types of care categorised by sex, was also observed in acute inpatient care, outpatient care and chronic and rehabilitation inpatient care, with expenditures of 9.76 million EUR, 2.28 million EUR and 1.28 million EUR for males, and 13.65 million EUR, 5.34 million EUR and 3.0 million EUR for females, respectively.

In terms of the distribution of expenditures by sex, we found that within the highest cost types of care, expenditures were distributed as follows: acute inpatient care: 41.7% male and 58.3% female; outpatient care: 29.9% male and 70.1% female; chronic and rehabilitation inpatient care: 29.8% male and 70.2% female, respectively. Overall, 36.8% of the costs were attributed to the treatment of male patients, while 63.2% were allocated to female patients. The distribution of expenditure mirrored that of the number of patients per NHIFA fund, as the number of female patients was higher, resulting in greater expenditure associated with their care in all treatment categories.

**Fig. 4:**
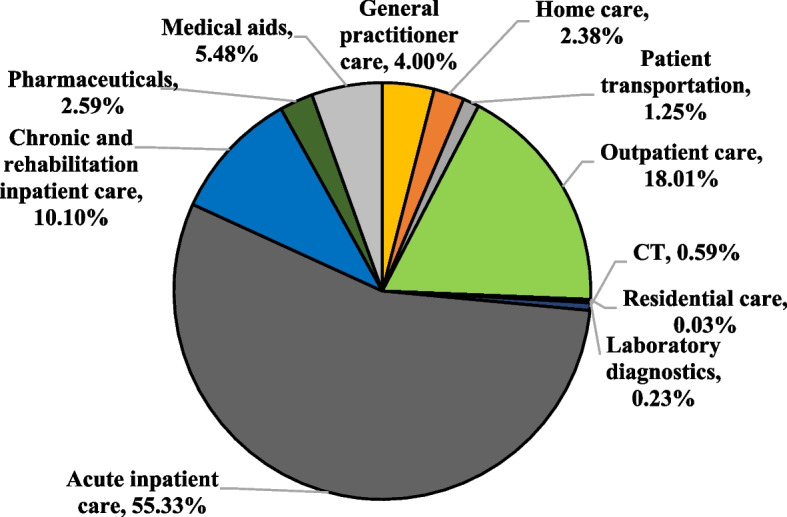
Distribution of NHIFA expenditure according to type of care in Hungary, 2018

There was a large difference in the expenditure of NHIFA funded treatments, as illustrated in Fig. 4. 55.3% of total NHIFA expenditure was in acute inpatient care, 18.0% in outpatient care and 10.1% in chronic and rehabilitation inpatient care, accounting for 62.7%, 14.6% and 8.2% of total health care expenditure for males, and 51.0%, 20.0% and 11.2% for females, respectively.

The proportion of all other categories was 16.6% (14.5% for males and 17.8% for females). During the study, we also assessed the proportion of the Health Insurance Fund by NHIFA treatment categories, which showed that home care accounted for 7.05% of total health insurance expenditure. This finding may be attributed to the high utilisation of physiotherapy during the rehabilitation period.

**Fig. 5:**
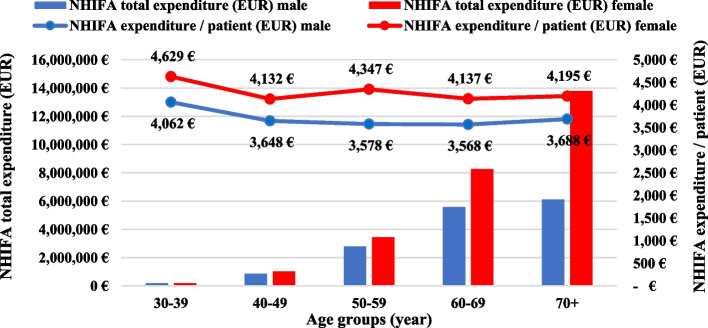
Total health insurance expenditure and average expenditure per patient by age group and sex based on acute inpatient care in Hungary, 2018

Figure 5 shows the total health insurance expenditure and average expenditure per patient categorised by age group and sex, based on acute inpatient care in Hungary. When examining the utilisation of the most costly acute inpatient care fund by age and sex, we found that the overall expenditure rate for females was substantially higher with age, particularly in the age group over 70. Among males, higher values were also seen with age (highest for those of 70 +), but at a lower rate. The extremely high expenditure of females over 70 (6.599 million EUR) represented 48.33% of total female patient expenditure and 28.19% of total expenditure for both sexes (Fig. 5). We also found that NHIFA (males, females) spent 46.99% (19.885 million EUR) of its total expenditure on the age group 70 + , 32.71% (13.839 million EUR) on the age group 60–69 and 14.71% (6.225 million EUR) on the age group 50–59, respectively. Among the age group 70 + years, females accounted for a high proportion of the age group expenditure, with 32.57%, 19.54% for the age group 60–69 years and 8.12% for the age group 50–59 years, respectively. We finally summarised that in 2018, the average total annual health insurance expenditure per patient treated for hip osteoarthritis was 3,966 EUR in total, with 3,627 EUR for males and 4,194 EUR for females. The average annual health insurance costs per patient were 15.6% higher for females.

**Fig. 6:**
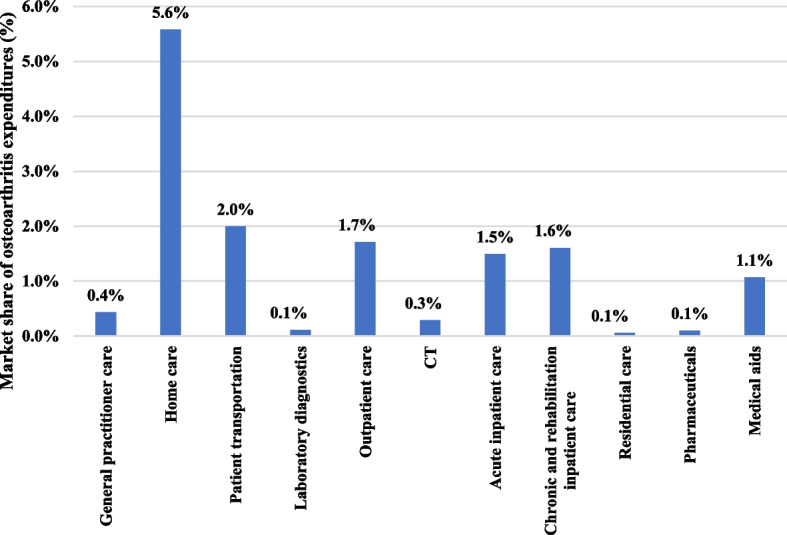
Market share of osteoarthritis expenditures from the total nationwide health insurance expenditures according to the different types of health care provisions

The cost of hip osteoarthritis for all types of health care provisions was compared to the total nationwide health insurance (NHIFA) expenditure for all diseases (Fig. 6). Therefore, we obtained the market share of hip osteoarthritis within the total nationwide NHIFA expenditure for each type of health care provision. Overall, hip osteoarthritis accounted for 1.0% of the total nationwide health insurance expenditures. And it varies among the 11 different types of health care provisions or settings ranging from 0.1% (laboratory diagnostics) to 5.6% (home care).

## Discussion

In this study, we conducted a nationwide epidemiological and health insurance disease burden analysis of hip osteoarthritis with ICD code M16 for the year 2018 in Hungary. While there are numerous international publications on the epidemiology and costs of hip osteoarthritis, no such study has been conducted in Hungary before [28, 29].

According to our results, the highest national patient numbers were found in outpatient care (217,686 patients), followed by general practice care (201,581 patients) and pharmaceuticals (97,689 patients). The number of patients was higher for females in all categories, which can be explained mainly by the sex differences in prevalence. International studies suggest that males may be less likely to seek health care for their problems, resulting in lower rates of medication prescription, GP visits, or rehabilitation treatment [30, 31].

The prevalence rates of each type of care varied considerably, with acute inpatient care having the lowest prevalence and outpatient care showing the highest prevalence. Therefore, it is crucial to consider the type of care utilised when calculating prevalence values. Based on patient numbers in outpatient care, the prevalence per 100,000 among men was 1,483.7 (1.5%), among women 2,905.5 (2.9%), in total 2,226.2 patients (2.2%). The age-standardised prevalence showed a similar pattern: 1,734.8 (1.7%) for males, and 2,594.8 (2.6%) for females per 100 000 population, for a total of 2,237.6 (2.2%). For both males and females, the highest number of patients was in the age group over 70 years, which means that the number of patients was higher with age. At this age group, the number of patients in males was considerably lower compared to females, but the prevalence rate remained high. This discrepancy may be explained by the lower average life expectancy of males. When we compared the acute inpatient patient numbers with the chronic and rehabilitation inpatient patient numbers, we observed that females over the age of 30 years had a higher rate of rehabilitation utilisation than males. In age groups 30–39, 40–49, 50–59, 60–69, and 70 + , the rehabilitation utilisation rate among female patients was 56.10%, 45.93%, 68.10%, 78.69%, and 98.20%, respectively, while among males, these rates were 34.04%, 33.62%, 42.44%, 53.17%, and 66.22%. Overall, 69.41% of females and 45.90% of males (in total 57.65%) over 30 underwent rehabilitation. *Ritter *et al*.* found that 82.6% of patients received early postoperative rehabilitation after total hip arthroplasty, based on data from the AOK Baden-Württemberg (Statutory Health Insurance). Younger people and males had lower utilisation of care, consistent with our results [32]. *Belay *et al*.* also reported in 2022 that patients over 70 years (49.3% over 70 years vs. 20.9% below 70 years) and females (58.7% for females vs. 46.8% for males) had significantly higher rates of rehabilitation utilisation [30]. We can also confirm these findings for patients over 70 (82.21% over 70 years vs. 51.51% under 70 years) and for females (69.41% for females vs. 45.90% for males).

The prevalence of hip osteoarthritis will substantially increase over the coming decades due to the ageing population in both developed and developing countries, and because of the risk factors associated with the development of OA (e.g. obesity and metabolic disease, sedentary lifestyle, age, gender, ethnicity and race, genetics, nutrition, smoking, bone density and muscle function) [1, 5, 33, 34].

In 2018, the NHIFA spent 42.31 million EUR on the treatment of hip osteoarthritis. A higher proportion of expenditure (63.2%) was attributed to female patients, and acute inpatient care was the main cost driver. The distribution of expenditure mirrored the number of patients per NHIFA funds, with higher patient numbers among females resulting in greater expenditure across all treatment categories. When examining the utilisation of the most costly acute inpatient care fund by age and sex, we found that the overall expenditure rate for females was substantially higher with age, especially in the age group over 70. The average annual treatment cost per patient was 3,966 EUR. The extremely high expenditure among females over 70 (6.599 million EUR) represented 48.33% of total female patient expenditure. While international studies also suggest a similar cost distribution between males and females, they are limited in scope and do not adequately explain the observed differences [35–37]. We can assume that female patients are more likely to pick up the presciption and to visit their doctor regularly. In Eastern European health care systems compared to Western Europe, there is a shift towards hospital care instead of outpatient or primary care. The differences in care structure and in the utilization might explain partly the differences [38, 39].

Many publications examining the epidemiological and health insurance disease burden of hip osteoarthritis have consistently reported that its prevalence increases gradually with age and is higher in females compared to males. *Van Saase *et al*.* made similar findings, describing that while males had a higher prevalence of the disease than females before the age of 50, females had a higher prevalence of the disease after the age of 50 [40]. *Litwick *et al*.* also confirmed the same results in 2013 in a study focusing on osteoarthrosis [1]. Additionally, a Peruvian disease burden study conducted by *Araujo-Castillo *et al*.* in 2016 highlighted the substantial disease burden of hip osteoarthritis, particularly among females and patients over 60 years of age [14]. Our study may confirm these claims, as we found a higher prevalence in females (1.96 times higher) and was higher with age based on outpatient care data.

According to results published by *Bijlsma *et al*.* in 2007, the prevalence of hip osteoarthritis was reported as 9.63 per 1,000 persons in males and 19.61 in females, based on data from the Dutch Institute for Public Health (RIVM) [41]. Our prevalence results based on outpatient care showed a higher rate of 14.84 per 1,000 people for males and 29.06 per 1,000 people for females.

*Fu et al.’s* results showed that the incidence of hip osteoarthritis in males is 1.93 times higher than in females, which in our case, the prevalence was 1.96 times higher for females than for males [10].

A systematic review of the prevalence of radiographic primary hip osteoarthritis in 2009 indicated a continuously increasing trend with aging. In the age groups 35–39, 40–44, 45–49, 50–54, 55–59, 60–64, 65–69, 70–74, 75–79, 80–84, and 85 + years, the prevalence was 1.6%, 0.7%, 1.7%, 2.0%, 3.5%, 4.8%, 6.4%, 8.3%, 10.1%, 9.9%, and 14.0%, respectively. According to the comparison of age-group prevalence rates for females and males, females had a higher prevalence rate in 6 out of 11 age groups [42]. Our results showed a prevalence of 0.2%, 0.8%, 2.7%, 5.0%, and 7.7%, respectively, in age groups 30–39, 40–49, 50–59, 60–69, and 70 + , also describing a continuously increasing trend. We found a higher prevalence of female patients in all 7 of the examined age groups. The prevalence values presented in age group distributions are consistent with those reported by Hirsch (1998) and Jacobsen (2004). *Hirsch *et al*.* found prevalence rates of 3.1%, 3.3%, 5.0%, and 4.8%, respectively, for the 45–54, 55–64, 65–74, and 75 + age groups [43]. *Jacobsen *et al*.* reported a prevalence of 2.7 in the 35–59 age group and 7.8 in the 60 + age group [44].

In the literature review, we found that published prevalence values vary considerably, which may be influenced by several factors. In addition to age and sex, ethnicity and geographical region also determines the prevalence of hip osteoarthritis. Many studies showed that Europe (12.59%) and North America (7.95%) had a higher prevalence of hip OA compared to Asia (4.26%) and Africa (1.2%) [2, 10–12, 42, 45].

The research methodology applied in the studies and the sources of the prevalence data also have a major influence on the value obtained. A Global Burden of Disease Study using global data from 2010 reported a prevalence of 0.85%, while a study from 2019 indicated a prevalence of 0.4%, compared to our nationwide result of 2.2% [8, 16]. Based on data from nationwide health insurance databases, *Kim *et al*.* described a prevalence of 1.2% in the population aged 71–95 (Health Insurance Review Agency (HIRA) database, South Korea) and *Araujo-Castillo *et al*.* observed a prevalence of 5.9% on a population aged 15 or older (Peruvian social health insurance system, EsSalud) [14, 46].

For data obtained from the databases, it is also important to determine the type of care under investigation. *Endres *et al*.* presented a prevalence of 6.1% (6.02% in men, 6.18% in women) among populations aged 40 years or older, based on outpatient and inpatient health insurance data (AOK Baden-Württemberg medical care data). Meanwhile, *Postler *et al*.* reported a prevalence of 6.2% according to outpatient data (5.8% in men, 6.5% in women) among populations aged 60 years or older (Germany statutory health insurance (BARMER) [47, 48]. Our results differed considerably by type of care (Fig. 1), with outpatient care showing a prevalence of 2.2%, pharmaceuticals 1%, acute inpatient care 0.1%, and general practice 2.1%.

*Odding *et al*.* conducted a home interview survey as part of the Rotterdam Study, involving a total population of 2,895 people aged 55 years and older, with an extremely high overall prevalence of 15.2% (14.1% male, 15.9% female) [49]. *Van Saase *et al*.* also found a high prevalence rate in their questionnaire survey involving 6,585 adults aged 45 years and older (13.7% in total, 12.7% male, 14.6% female) [40]. Conducted a similar interview-based questionnaire survey, *Andrianakos *et al*.* reported a lower prevalence (0.9% in total, 0.3% male, 1.5% female) among a population of 8,740 people aged 19 years and older [50].

There are also different classifications according to the definition of osteoarthrosis, which may also influence the prevalence value obtained. *Jordan *et al*.* showed a prevalence of 27.6% (25.4% male, 29.5% female) for radiographic hip osteoarthritis and 9.7% (8.3% male, 11.1% female) for symptomatic hip osteoarthritis [51]. *Kim *et al*.* measured a prevalence of 19.6% (24.7% male, 13.6% female) for radiographic hip osteoarthritis and 4.2% (5.2% male, 3.0% female) for symptomatic osteoarthritis [52]. *Iidaka *et al*.* reported results of 15.7% (18.2% male, 14.3% female) and 0.75% (0.29% male, 0.99% female), respectively [53]. In a self-report study, *Plotnikoff *et al*.* found a prevalence of 8.5% (4.4% male, 7.6% female) [54]. All these factors (e.g. age, sex, geographical location, research type, methodology, sample size), may all influence and bias the prevalence outcomes, which makes it particularly important to critically evaluating the origins of our results.

Hip osteoarthritis imposes not only a burden on society but also on the national economy. *Le Pen *et al*.* (COART study) examined the cost distribution for the treatment of osteoarthritis, where chronic and rehabilitation inpatient care (11%) and acute inpatient care (38%) had similar cost ratios to our results for hip osteoarthritis (10%, 55%). In France, the total cost of osteoarthritis treatment exceeded 1.6 billion EUR in 2002, while in Hungary, the expenditure solely for hip osteoarthritis treatment in 2018 amounted to 42.31 million EUR. France allocated 570 million EUR to pharmaceuticals and 820 million EUR to inpatient care, whereas our expenditures for hip osteoarthritis treatment were 1.098 million EUR (pharmaceuticals) and 27.684 million EUR (acute and chronic inpatient care) [55]. A recent study on the cost of osteoarthritis suggested that the annual cost per person was around 10,000 EUR (10,800 USD) [19]. According to a Japanese cross-sectional study by *Ebata-Kogure *et al*.,* most patients were treated in outpatient care for hip osteoarthritis (consistent with our findings), and the per capita annual median healthcare cost was estimated at 35,000 JPY [56]. *Malik *et al*.* estimated that the cost of care associated with hip osteoarthritis ranged from 500$ to 800$ per patient in the year before hip replacement surgery [20].

For health insurance disease burden studies, in addition to direct costs (e.g. cost of surgery, hospital resources, caregiver time, pharmacological and non-pharmacological treatment), it is important to identify indirect costs (e.g. absenteeism, costs due to loss of income, loss of productivity, early retirement, premature mortality, expenditure on home care, disability payments), which were not included in this study. According to *Bitton *et al*.* indirect costs could reach as high as 4,600 USD per person annually [17]. The cost of indirect absenteeism was estimated by *Kotlarz *et al*.* at around $10.3 billion in the United States [35]. According to a recent Dutch study by *Hardenberg *et al*.*, an average sick leave episode due to hip osteoarthritis lasted 159 calendar days and cost €12,482. These costs were particularly high for male workers and those working more hours per week. The average annual expenditure on sick leave due to hip osteoarthritis was €13.8 million for the Dutch workforce [57].

Comparing our findings with a Hungarian study (*Gazsó *et al*.*) also using NHIFA data from 2018 on pertrochanteric fracture, we observe that the prevalence per 100,000 population was also lower in males (51.1 patients) than in females (114.7 patients). While the total health insurance expenditure for pertrochanteric fractures was 22.98 million EUR, the total health insurance expenditure for hip osteoarthritis was 42.31 million EUR. A significant part of the expenditure was also incurred by female patients and in acute inpatient care [58, 59].

The limitations of the study may include, that it covered a period of one year (2018), so no long-term conclusions can be made regarding trends. To eliminate possible duplications across different health insurance funds, the outpatient fund with the largest patient numbers was used to determine the national prevalence data, and the most costly acute inpatient care was used to define the annual health insurance expenditure and per capita expenditure. An important factor in the results of our data analysis is that the data provided by NHIFA is our only national and comprehensive source; there is no alternative data system, such as a hip osteoarthritis register, so currently only the analysis of NHIFA data can offer a comprehensive national perspective on the epidemiological and health insurance burden. Only direct costs as reported by NHIFA were examined, indirect costs were not included in our analysis, which could be considered for future research. In the lack of individual patient level data we were not able to conduct statistical tests, which may be a weakness of our research. Furthermore, it is important to emphasise that the database only included patients who had accessed the public health care system. Patients who experienced symptoms but did not seek medical care were not included in the database. It is also possible that those who had symptoms went to private health care, which can also lead to bias.

Our nationally representative study is a gap-filling study on hip osteoarthritis, as the disease represents an extremely high health, social and economic burden. The limited availability of national data and research justifies its investigation from an epidemiological and health insurance perspective. Our research presents important data for the health care systems and society, that can help inform health policy making, in the preparation of social insurance decisions, and in the formulation of care and prevention strategies. Our research findings can be applied to health care management and clinical practice in several ways. Results can be useful for outpatient departments in the management of consultation times and appointment scheduling, and therefore improving ambulatory patient flow management. It can also be used in the management of hip replacement surgery to reduce long waiting lists, facilitating better planning of healthcare capacity, including operating room resources. Moreover, it can also be applied for planning rehabilitation capacities after surgery, ensuring adequate support for patients during their recovery journey. By leveraging these insights, healthcare providers can deliver more efficient and effective care, ultimately enhancing the overall patient experience and contributing to more optimal patient care.

## Conclusions

Hip osteoarthritis is a highly prevalent chronic joint disorder that constitutes a significant and growing burden on patients, health care systems and the broader society. Overall, our main findings showed that the crude prevalence of the disease was 1.96 times higher (the age-standardised prevalence was 1.5 times higher) in females than in males and was considerably higher with age. The global rise in prevalence is largely attributed to factors such as an ageing population, increased physical workload, obesity, and adverse lifestyle factors. According to analyses of the health insurance disease burden, acute inpatient care was the major cost driver in the treatment of hip osteoarthritis. Distribution of major cost drivers showed a substantial difference between the sexes. The average annual health insurance costs per patient were 15.6% higher for females.

Epidemiological studies have often reported diverse prevalence results, which may be attributed to various factors including differences in the origin of the data used, differences in age, sex, geographical location, research type, methodology and sample size investigated. To ensure comparability and relevance of results, it is important that all these factors are clearly defined.

In conclusion, the analysis of the disease burden in Hungary on nationwide data showed that the annual health insurance expenditure for hip osteoarthritis is very high. This aspect also confirms the importance of the social burden of hip osteoarthritis from a health economics perspective, emphasizing the importance of interventions, education, and prevention in this area.

## Data Availability

The datasets used and/or analysed during the current study are available from the corresponding author on reasonable request.

## Abbreviations

OA: Osteoarthritis
ICD: International Classification of Diseases
GP: General practitioner
CT: Computed tomography
MRI: Magnetic resonance imaging
PET: Positron emission tomography
NHIFA: National Health Insurance Fund Administration
HCSO: Hungarian Central Statistical Office
DALY: Disability adjusted life years
ADL: Activities of daily living
WHO: World Health Organization
ICPM: International Classification of Procedure in Medicine
DRG: Diagnosis-related group
OECD: Organisation for Economic Co-operation and Development
